# The Influence of Planning and Response Inhibition on Cognitive Functioning of Non-Psychotic Unipolar Depressed Suicide Attempters

**DOI:** 10.5964/ejop.v13i4.1385

**Published:** 2017-11-30

**Authors:** Marco Moniz, Saul Neves de Jesus, Andreia Pacheco, Eduardo Gonçalves, João Viseu, Marta Brás, Dina Silva, Sílvia Batista

**Affiliations:** a Department of Psychiatry and Mental Health, Hospital Center of Algarve, Faro, Portugal; b Faculty of Human and Social Sciences, University of Algarve, Faro, Portugal; c Research Centre for Spatial and Organizational Dynamics (CIEO), Faro, Portugal; d Algarve Biomedical Center (ABC), Faro, Portugal; Webster University Geneva, Geneva, Switzerland; Glasgow Caledonian University, Glasgow, United Kingdom

**Keywords:** unipolar depression, cognitive functioning, executive dysfunction, planning, response inhibition, suicide attempts

## Abstract

Depression is one of the main risk factors for suicide. However, little is known about the intricate relationships among depressive symptomatology in unipolar depression, suicide risk, and the characteristics of executive dysfunction in depressed patients. We compared 20 non-psychotic unipolar depressed suicide attempters to 20 matching depressed non-attempters and to 20 healthy controls to further investigate the possible differences in neuropsychological performance. Depressed subjects were controlled for current suicidal ideation, and their neuropsychological profile was assessed using a range of measures of executive functioning, attention, verbal memory, processing speed, and psychomotor speed. Depressed groups were outperformed by healthy controls. Depressed attempters presented more cognitive impairment than depressed non-attempters on a simple Go/No-go response inhibition task and performed better than non-attempters on the Tower of London planning task. Depressed attempters were clearly distinguished by a deficit in response inhibition (Go/No-go commission errors). The normative planning performance (Tower of London extra moves) of the suicide attempters was unexpected, and this unanticipated finding calls for further research. Normative planning may indicate an increased risk of suicidal behavior.

Suicide is a global phenomenon. According to the World Health Organization (WHO, 2015), more than 800,000 individuals commit suicide every year. In 2012, suicide represented the second leading cause of death among people aged 15-29 years old worldwide. Mental disorders, particularly depression and alcohol abuse, are among the main risk factors for suicide (Schneider et al., 2006; WHO, 2015). A meta-analysis performed by Arsenault-Lapierre, Kim, and Turecki (2004) provides evidence of mental disorders in 87.3% of suicide completers, 95% of whom meet the criteria for affective disorders. Individuals suffering from such disorders continue to be stigmatized in many societies (WHO, 2015). Consequently, such individuals tend to underrate or conceal their symptoms when they are aware of them and prefer to isolate themselves rather than seeking help. This behavior hinders early diagnosis and further complicates the already difficult pursuit of adequate response and control. Thus, healthcare professionals must increasingly identify suicide causes among these patients to predict and prevent it.

Nevertheless, little is known about the real association between depression and suicide risk. For instance, Jollant, Lawrence, Olié, Guillaume, and Courtet (2011) posit that the pathological processes underlying a crisis usually occur during an acute stage of the illness. By contrast, Keilp et al. (2012) suggest that the link between suicide risk and the severity of depression is a modest one. Furthermore, in depression, the relationship between suicidal ideation and suicide attempts also remains controversial. Whereas some depressed patients have never entertained suicidal thoughts or attempted suicide (Marzuk, Hartwell, Leon, & Portera, 2005), more than half of such patients contemplate it, and one-third of ideators commit it (Kessler, Borges, & Walters, 1999). Consequently, although depression is considered a high risk factor for suicide (Schneider et al., 2006), this high risk may not be directly associated with the psychopathological condition itself because ideation and acts represent distinct stages of the suicidal process (Jollant et al., 2011). Alone, ideation is a weak predictor of attempts (Jollant et al., 2011; Marzuk et al., 2005). Recent epidemiological findings have suggested that depression is a better predictor of suicidal ideation than of suicidal acts (Nock, Hwang, Sampson, & Kessler, 2010). Therefore, uncovering markers that could allow these two stages of suicidal behavior to be more clearly distinguished is highly significant for early diagnosis, treatment, and prevention.

In recent years, research ranging from original studies to evidence-based review articles has aimed to identify neuroimaging and cognitive markers in clinical samples of patients with a history of suicide attempts and/or current suicidal ideation. Regarding the neuroanatomical underpinnings of suicidal behavior, a recent meta-analysis identifies the involvement of the ventrolateral prefrontal cortex (VLPFC), including regions of the orbitofrontal cortex (OFC), the anterior cingulate gyrus, the dorsomedial PFC (DMPFC), the dorsolateral PFC (DLPFC), and, to a lesser extent the amygdala and the medial temporal cortex (MTC) (Jollant et al., 2011). Some of these regions, e.g., the DLPFC, the anterior cingulate cortex (ACC), and the OFC, are believed to participate in basically all executive functions (EFs) (Rogers et al., 2004). EFs refer to cognitive competences that allow individuals to determine objectives in changing environments and to find ways of meeting them by constantly adapting themselves to their circumstances (Burgess & Alderman, 2003). According to previous reports, executive dysfunctions, which are different from other cognitive deficits common in depression, are linked to suicidal behavior (Jollant et al., 2011; Keilp, Gorlyn, Oquendo, Burke, & Mann, 2008; Keilp et al., 2001, 2013; King et al., 2000; Marzuk et al., 2005; Richard-Devantoy, Berlim, & Jollant, 2014; Richard-Devantoy, Gorwood, et al., 2012; Richard-Devantoy, Jollant, et al., 2012; Westheide et al., 2008). Corroborating most neuroimaging findings, research on cognitive markers has found alterations in the EFs of depressed suicide attempters (Jollant et al., 2011; Keilp et al., 2001, 2008, 2013; Marzuk et al., 2005; Richard-Devantoy, Gorwood, et al., 2012; Richard-Devantoy, Jollant, et al., 2012; Richard-Devantoy et al., 2014; Westheide et al., 2008), particularly regarding attention, problem solving, verbal fluency and mental flexibility, though this evidence is not always consistent across samples.

In a review, Richard-Devantoy, Gorwood, et al. (2012) find a strong connection between depression and executive dysfunction, suggesting that the two disorders may have the same neuropsychological basis in frontal dysfunction, which may enhance suicide risks. Many of these alterations in EFs correspond to some of the most common markers presented by depressed non-attempters, regardless of their current suicidal ideation (Beats, Sahakian, & Levy, 1996; Biringer et al., 2005; Lee, Hermens, Porter, & Redoblado-Hodge, 2012; McDermott & Ebmeier, 2009; Richard-Devantoy, Gorwood, et al., 2012; Richard-Devantoy, Jollant, et al., 2012; Richard-Devantoy et al., 2014; Rogers et al., 2004; Roiser, Rubinsztein, & Sahakian, 2009; Stordal et al., 2004; Wagner, Doering, Helmreich, Lieb, & Tadić, 2012). Moreover, Marzuk et al. (2005) and Westheide et al. (2008) suggest that suicidal ideation may contribute to the aggravation of EF impairment. Lee et al. (2012) attribute suicidal ideation to clinical state impairments in psychomotor speed and memory functioning, whereas deficits in attention and EFs are persistent (even after remission) and consequently are more prone to be trait-markers. According to Biringer et al. (2005), trait-related impairments may be associated with inherent pathobiological mechanisms.

Many recent studies have specifically assessed samples of depressed suicide attempters. Some studies examine unipolar and bipolar subjects (e.g., Keilp et al., 2001, 2008; Marzuk et al., 2005), whereas others examine patients with comorbid personality disorders (e.g., Keilp et al., 2013). However, only a few focus specifically on non-psychotic unipolar depressed patients (e.g., King et al., 2000; Richard-Devantoy, Jollant, et al., 2012; Westheide et al., 2008).

King et al. (2000), Richard-Devantoy, Jollant, et al. (2012), and Westheide et al. (2008) measure the performance of non-psychotic unipolar depressed attempters via several neuropsychological measures. The first two focus on older patients (older than 50 and older than 65, respectively), and only the third controls for current suicidal ideation. Regarding performance results on a range of neuropsychological tasks, King et al. (2000) compare depressed suicide attempters to depressed non-attempters. They observe the effects of aging on mental sequencing and flexibility, but they find no significant differences between the two groups. Conversely, a study by Richard-Devantoy, Jollant, et al. (2012) shows that elderly depressed attempters performed more poorly than matching depressed non-attempters and healthy controls in terms of cognitive inhibition. Westheide et al. (2008) find differences between healthy controls and depressed suicide attempters with and without current suicidal ideation. Moreover, their results reveal suicidal ideation to be related to more severe executive deficits, particularly in decision making (Westheide et al., 2008).

However, the hypothesis that suicide attempters and/or suicidal ideators can be distinguished by the presence of such unique executive impairments is far from being well established. For instance, King et al. (2000) challenge this hypothesis, as their results reveal no significant differences between depressed suicide attempters and non-attempters on any neuropsychological measures. So far, research has not been able to characterize depressed suicide attempters with a specific cognitive profile (Westheide et al., 2008) or to clearly distinguish them from depressed non-attempters, and the same applies to suicidal ideators in relation to non-ideators. Consistent findings remain scarce in view of the various questions that have yet to be definitively answered unclear. Therefore, more studies are needed to realize more effective suicide prevention and to contribute to a deeper understanding of suicidal behavior and the underlying processes that lead to suicidal acts.

The current study aims to further investigate differences in the neuropsychological performance of three adult age groups: non-psychotic unipolar depressed suicide attempters, non-psychotic unipolar depressed non-attempters, and healthy controls. Following King et al. (2000), Richard-Devantoy, Jollant, et al. (2012), and Westheide et al. (2008), we circumscribe our clinical sample to non-psychotic unipolar depression because the cognitive functions of bipolar subjects are believed to be influenced in a distinctive way (Stordal et al., 2004). Likewise, psychotic patients tend to exhibit more deficits (Stordal et al., 2004). Patients are controlled for current suicidal ideation, and other clinical parameters are applied to confirm diagnoses. We conduct a detailed neuropsychological assessment that includes tests of executive functioning, attention, verbal memory, processing speed, and psychomotor speed.

In light of previous reports, we hypothesize the following: (1) depressed subjects (attempters and non-attempters) will perform more poorly than healthy controls, and (2) depressed suicide attempters will generally exhibit more executive deficits, than depressed non-attempters and healthy controls.

## Methods

### Participants

We initially selected 51 patients from the outpatient clinic of the Department of Psychiatry and Mental Health of the Hospital Center of Algarve, and 11 subjects were excluded during the evaluation period. The control group was recruited through advertising. All subjects completed a battery of tests within one week, for a total of two sessions. A randomized system was used to order the test application. The sample was collected between February and August of 2013.

All three groups—two experimental groups (depressed suicide attempters, with at least one past suicide attempt, and non-attempting patients) and the control group—had 20 subjects each. The non-psychotic unipolar depressed suicide attempters group included 18 women and 2 men, with a mean age of 42.2 years (*SD* = 15.12) and a mean of 9.5 (*SD* = 3.68) years of education. The matching depressed non-attempter group included 13 women and 7 men, with a mean age of 44.3 years (*SD* = 14.78) and a mean of 8.9 (*SD* = 3.54) years of education. Each past attempter had a mean of 3 suicidal acts. Fifteen subjects used medication to attempt suicide (75 percent), and 5 committed a more violent suicidal act (e.g., hanging, gas inhalation). The group of healthy controls with analogous characteristics included 13 women and 7 men, with a mean age of 43.3 years (*SD* = 14.88) and a mean of 9.4 (*SD* = 3.61) years of education. All participants were Caucasian, right handed, and Portuguese speakers.

### Measures

In this study, we used computerized and standard paper-and-pencil instruments. We employed the following computer-administered instruments from the Psychology Experiment Building Language (PEBL) Test Battery (Mueller & Piper, 2014):

#### Go/No-Go Task (GNG)

GNG is one of the most useful measures for assessing response inhibition, where a motor response must be either executed or inhibited (Bezdjian, Baker, Lozano, & Raine, 2009). We applied the “simple” paradigm in which the no-go stimulus was always the same. The Portuguese version (Moniz, De Jesus, Gonçalves, Viseu, Pacheco, & Moreira, 2016) of the computerized GNG from the PEBL was used (Mueller & Piper, 2014), developed and validated previously by Bezdjian, Baker, Lozano, and Raine (2009). The GNG demonstrates reasonable test–retest reliability (Kindlon, Mezzacappa, & Earls, 1995).

#### Tower of London Task (TOL)

Inspired by Tower of Hanoi, a classic problem-solving puzzle (Kaller, Unterrainer, Rahm, & Halsband, 2004; McKinlay & McLellan, 2011; Newman, Greco, & Lee, 2009), TOL is a visuospatial planning task (Kaller et al., 2004; McKinlay & McLellan, 2011; Newman et al., 2009). We used the Portuguese version (Moniz, De Jesus, Viseu, Gonçalves, Pacheco, & Baptista, 2016) of the computerized TOL from PEBL (Mueller & Piper, 2014), which does not differ from the manual versions in terms of the level of difficulty (McKinlay & McLellan, 2011). The reliability and validity study was demonstrated by Piper et al. (2015), with a correlation coefficient of .36.

#### Iowa Gambling Task (IGT)

IGT aims to simulat real-life judgment alterations, allowing assessment of the subject’s emotions associated with decision making (Bechara, Damasio, Damasio, & Anderson, 1994). The participants were instructed to perform a series of 100 selections from a group of four decks of cards (A, B, C and D). The Portuguese version (Moniz, De Jesus, Gonçalves, Pacheco, & Viseu, 2016) of the computerized IGT from PEBL (Mueller & Piper, 2014) was used. The reliability and validity study was demonstrated by Piper et al. (2015), with a correlation coefficient of .41.

#### Victoria Stroop Test (VST)

Inhibitory control is an important cognitive function associated with executive functioning, which is assessed with tasks such as Stroop (Miyake et al., 2000). Our study employed the Portuguese version (Moniz, De Jesus, Gonçalves, Viseu, Baptista, & Pacheco, 2016) of the computerized VST from PEBL (Mueller & Piper, 2014), which is a briefer and slightly different version from the traditional one. The original version of VST (Troyer, Leach, & Strauss, 2006) revealed adequate psychometric data, including reliability and validity (Strauss, Sherman, & Spreen, 2006).

#### Wisconsin Card Sorting Test (WCST)

WCST is one of the most recurrently used neuropsychological measures to assess executive functioning, and it is commonly utilized to assess set maintenance and set shifting (Carrillo-de-la-Peña & García-Larrea, 2007). The participants were expected to place cards in one of four piles according to the characteristics of the stimuli. The Portuguese version (Moniz, De Jesus, Viseu, Gonçalves, Moreira, & Pacheco, 2016) of the computerized WCST from PEBL (Mueller & Piper, 2014) was applied. The reliability and validity study was demonstrated by Piper et al. (2015), with a correlation coefficient of .45.

#### Finger Tapping Task (FTT)

FTT assesses fine motor speed and motor control (Christianson & Leathem, 2004). The Portuguese version (Moniz, De Jesus, Pacheco, Gonçalves, & Viseu, 2016) of the computerized FTT from PEBL (Mueller & Piper, 2014) was used (study that validated this version of the FFT), with the left and right index fingers: five consecutive trials for each hand, with a 10-second break following each trial and a 30-second break every five trials. The average number of taps over five trials was calculated for each hand. The reliability and validity studies of the computerized FTT were demonstrated by Hubel, Reed, Yund, Herron, and Woods (Hubel, Reed, Yund, Herron, & Woods 2013; Hubel, Yund, Herron, & Woods, 2013).

For computerized tests, a computer that ran Microsoft Windows 8.1 and contained a touch screen (IGT, TOL, WCST), a keyboard (VST), or an external keypad (FTT and GNG) was used. The paper-and-pencil instruments used were as follows:

#### Trail Making Test (TMT)

TMT comprises two parts—trials A and B. Trial A of TMT assesses attention, visual scanning, and information processing (Cavaco et al., 2008a, 2013a). The participants were given two sets of dots targeting numbers and were expected to connect them in sequential order (e.g., 1–2–3). Trial B assesses working memory and EFs, such as the capability to switch between sets of stimuli (Cavaco et al., 2008a, 2013a). For trial B, the participants were given two sets of 25 dots, one corresponding to numbers (1–13) and the other corresponding to letters (A–L) in sequential order. The sequence began with the first number, followed by the first letter alphabetically, then the second number, and so on (e.g., 1–A–2–B–3). We used the Portuguese version (Cavaco et al., 2013a).

#### Verbal Fluency Test (VFT)

In addition to EFs, VFT measures non-motor processing speed and language production, which recruit PFC and temporal lobes (Cavaco et al., 2013b). VFT consists of two tasks, namely, semantic and phonemic fluency. The subjects were asked to name as many animals as possible in 60 seconds and to say as many words beginning with M, R, and P in 60 seconds (for each letter), as described in the norms of the Portuguese version (Cavaco et al., 2013b).

#### Auditory Verbal Learning Test (AVLT)

Considered a measure that is sensitive to verbal memory deficits and neurological impairment, AVLT evaluates memory and verbal learning (Cavaco et al., 2008b) through five consecutive trials. After a 30-minute break, the participants were expected to recall the words that included the original one from a longer list. The Portuguese version (Cavaco et al., 2008b) was used.

### Procedures

All participants were assessed by a psychologist specifically certified for this purpose. Each participant completed a health and demographic questionnaire. During the selection of the sample, depression diagnoses were confirmed using the MINI International Neuropsychiatric Interview (MINI) (Sheehan et al., 1997) and the Portuguese version of the Brief Symptom Inventory (BSI) (Canavarro, 2007). Depressive symptomatology at the time of the assessment was evaluated using the 17-item Hamilton Depression Rating Scale (HAM-D-17) (Sousa, Lopes, & Vieira, 1979). To screen for the presence of personality disorders, the PDQ-4 + Personality Diagnostic Questionnaire (Hyler, 1994) was applied. Current suicidal ideation was assessed using the Portuguese version of the Suicidal Ideation Questionnaire (SIQ) (Ferreira & Castela, 1999). The Rey 15-Item Memory Test (15-IMT) was employed to detect malingering (Simões et al., 2010).

Exclusion criteria were current or prior bipolar disorder, schizophrenia, major psychosis, dementia, substance abuse, personality disorder, neurologic disease (including head injury involving a loss of consciousness), and suspected malingering.

This study was approved by the Hospital Center of Algarve Ethics Committee and was in according with the principles of the Declaration of Helsinki. After being provided with all the information about the study, all the participants signed an informed consent form. For ethical reasons, we could not assess the clinical sample off medication.

All analyses were conducted using the Statistical Package for Social Sciences (SPSS), version 20.0. The level of significance was set at *p* < than .05. The normality of distribution for continuous variables was tested with the Shapiro-Wilk test. A *χ^2^* test was used to compare categorical variables, and a *t*-test (for two groups) or analysis of variance (ANOVA) (for more than two groups) was used to compare continuous variables. Tukey’s procedure and Bonferroni correction were used for pairwise comparisons.

## Results

### Group Characteristics

A one-way ANOVA revealed that depressed groups (attempters and non-attempters) and healthy controls did not differ significantly with regard to age (*F*(2,57) = .738, *p* = .483, $\eta_{p}^{2}$ = .025), gender (*χ^2^*(2, *N* = 50) = 4.261, *p* = .119), or education (*F*(2,57) = 1.658, *p* = .200, $\eta_{p}^{2}$ = .055) (Table 1).

**Table 1:** Descriptive Statistics (N = 60)

Measures	Depressed non-attempters^a^	Depressed suicide attempters^b^	Healthy controls^c^	*p*-value	*Post Hoc* Tukey
	*M (SD)*	*M (SD)*	*M (SD)*		
Social and Clinical Measures					
Age, Years	44.28 (14.78)	42.22 (15.12)	43,25 (14,88)	.483	---
Education	8.94 (3.54)	9.53 (3.68)	9,36 (3,61)	.200	---
BSI-D, Score	2.26 (.98)	2.44 (1.18)	---	.611	---
HAM-D-17, score	17.20 (7.33)	20.42 (7.59)	---	.186	---
Cognitive Measures					
VFT-Semantic Fluency-Animals	14.0 (3.44)	14.94 (3.68)	---	.420	---
VFT-Phonemic Fluency- (M, R, P)	21.50 (8.0)	21.76 (6.55)	---	.914	---
AVLT-Immediate Recall	46.1 (9.0)	50.63 (8.53)	---	.321	---
AVLT-Delayed Recall (30’)	9.8 (2.28)	10.57 (2.54)	---	.117	---
AVLT-Memory Retention	85.42 (10.0)	87.44 (14.64)	---	.617	---
TMT-Part A	57.03 (15.9)	54.61 (18.03)	34.07 (14.74)	.000	HC ˂ NA, SA
TMT-Part B	154.45 (54.16)	131.71 (72.48)	72.92 (32.49)	.001	HC ˂ NA, SA
FTT-Dominant Hand	53.15 (7.88)	53.66 (8.71)	62.45 (7.63)	.001	HC ˃ NA, SA
FTT-Non-Dominant Hand	46,15 (7.29)	46.72 (8.44)	52.40 (6.45)	.018	HC ˃ NA
TOL-Extra Moves	22.52 (8.59)	17.78 (6.77)	16.0 (7.24)	.028	NA ˃ SA, HC
TOL-Time	456.7 (204.4)	366.5 (117.4)	267.7 (63.96)	.000	HC ˂ NA
IGT-Net Score	8.31 (23.23)	17.22 (17.41)	33.22 (23.57)	.005	HC ˃ NA
WCST-CAT (Moves To Complete 1^st^ Category)	24.26 (23.76)	27.10 (22.31)	14.55 (5.26)	.127	---
WCST-PE (Preservative Errors)	19.89 (10.85)	19.0 (10.89)	8.61 (6.15)	.001	HC ˂ NA, SA
WCST-CLR (Conceptual-Level Responses)	59.03 (16.37)	54.65 (21.68)	78.05 (11.17)	.000	HC ˃ NA, SA
VST-Errors	3.41 (3.46)	3.47 (3.53)	1.31 (1.82)	.055	---
VST-C Time	119.9 (76.25)	100.44 (76.74)	62.77 (23.61)	.028	HC ˂ NA
GNG-CE (Commission errors)	6.0 (4.0)	11.95 (6.15)	6.0 (2.93)	.000	SA ˃ NA, HC
GNG-OE (Omission Errors)	.90 (1.48)	.55 (.88)	.15 (.36)	.075	---
Reaction Time, ms (Go)	454.9 (86.1)	457.1 (90.0)	437.4 (65.1)	.704	---
Reaction Time, ms (No-Go)	593.5 (84.7)	553.1 (44.8)	538.4 (54.3)	.023	NA ˃ HC

*Note.* NA = depressed Non-Attempters; SA = depressed Suicide Attempters; HC = Healthy Controls; BSI-D = Depression scale from Brief Symptom Inventory; HAM-D-17 = Hamilton Depression Rating Scale - 17 items; VFT = Verbal Fluency Test; AVLT = Auditory Verbal Learning Test; TMT = Trail Making Test; FTT = Finger Tapping Task; TOL = PEBL Tower of London; IOWA = PEBL Bechara (Iowa) Gambling Task; WCST = PEBL Wisconsin (Berg) Card Sorting Test.

^a^*n* = 20. ^b^*n* = 20. ^c^*n* = 20.

The *t*-tests revealed no significant differences between depressed groups with respect to depressive symptoms (BSI-D [*t*(38) = -.512, *p* = .611, *d* = -.172], HAM-D-17 [*t*(38) = -1.348, *p* = .186, *d* = -.431]), current suicidal ideation (SIQ Total [*t*(38) = 1.611, *p* = .115, *d* = .509]), or suicidal intent (SIQ, Item 18 [*t*(38) = .312, *p* = .757, *d* = .099]).

### Neuropsychological Performance

In the GNG task, we found differences among the three groups in terms of commission errors (*F*(2,57) = 11.315, *p* ˂ .001, $\eta_{p}^{2}$ = .284) and no-go reaction time (*F*(2,57) = 4.016, *p* = .023, $\eta_{p}^{2}$ = .124). Still, in terms of commission errors, attempters performed worse than depressed non-attempters and the control group, and no significant differences were found between depressed non-attempters and healthy controls (*t*(38) = 0, *p* = 1, *d* = 0). In the TOL, only depressed non-attempters performed worse. Differences were found among the three groups in terms of extra moves (*F*(2,55) = 3.839, *p* = .028, $\eta_{p}^{2}$ = .122) and performance time (*F*(2,55) = 8.988, *p* < .001, $\eta_{p}^{2}$ = .246), and no differences were found between depressed attempters and healthy controls (*t*(37) = .796, *p* = .431, *d* = .253).

Depressed patients (attempters and non-attempters) were outperformed by healthy controls in almost all measures, with differences between the three groups in IGT (*F*(2,49) = 5.906, *p* = .005, $\eta_{p}^{2}$ = .194), VST errors (*F*(2,52) = 3.074, *p* = .055, $\eta_{p}^{2}$ = .106), VST c-time (*F*(2,52) = 3.819, *p* = .028, $\eta_{p}^{2}$ = .128), WCST perseverative errors (*F*(2,53) = 7.798, *p* = .001, $\eta_{p}^{2}$ = .227), the percentage of conceptual-level WCST responses (*F*(2,53) = 9.758, *p* < .001, $\eta_{p}^{2}$ = .269), FTT dominant hand (*F*(2,55) = 8.165, *p* = .001, $\eta_{p}^{2}$ = .229), FTT non-dominant hand (*F*(2,55) = 4.301, *p* = .018, $\eta_{p}^{2}$ = .135), TMT-A (*F*(2,51) = 9.171, *p* < .001, $\eta_{p}^{2}$ = .265), and TMT-B (*F*(2,51) = 8.558, *p* = .001, $\eta_{p}^{2}$ = .255).

**Figure 1:**
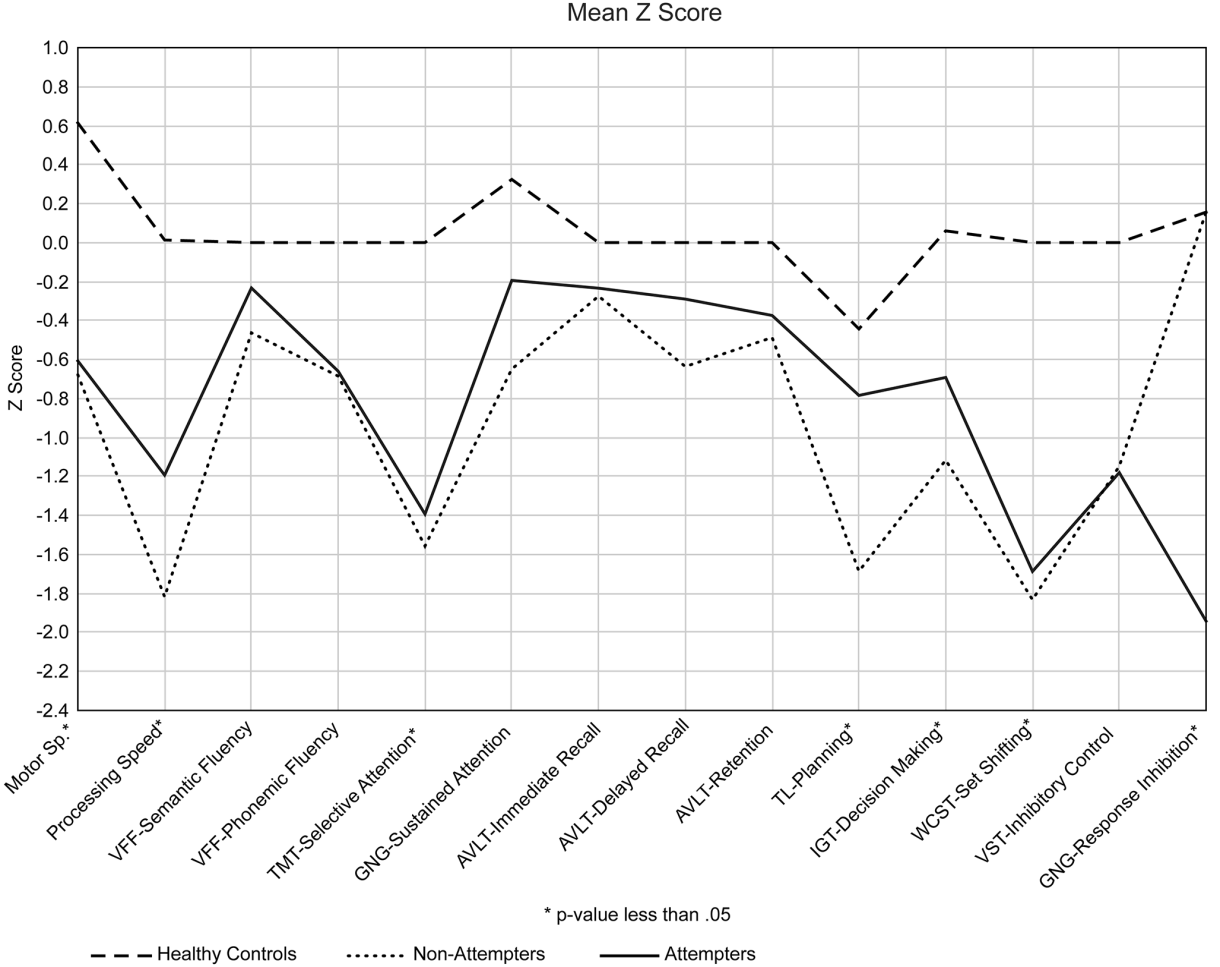
Z scores of the neuropsychological tests presented by each cognitive domain.

Compare only the depressed groups, we did not find significant differences in IGT (*t*(32) = -1.274, *p* = .212, *d* = -.434), VST errors (*t*(34) = -.053, *p* = .958, *d* = -.017), WCST perseverative errors (*t*(36) = .254, *p* = .801, *d* = .081), the percentage of conceptual-level WCST responses (*t*(36) = .703, *p* = .487, *d* = .228), FTT dominant hand (*t*(36) = -.190, *p* = .850, *d* = -.060), FTT non-dominant hand (*t*(36) = -.224, *p* = .824, *d* = -.632), TMT-A (*t*(38) = .449, *p* = .656, *d* = .142), TMT-B (*t*(37) = 1.113, *p* = .273, *d* = .355). When we isolated only the attempters and compared them based in terms of the type of suicidal act (violent or non-violent), we did not find significant differences, particularly with regard to the GNG task, commission errors (*t*(18) = .143, *p* = .888, *d* = .063), TOL task (*t*(17) = -1.072, *p* = .299, *d* = -.551), and IGT (*t*(16) = -.546, *p* = .593, *d* = -.390). For VFT and AVLT, we did not assess healthy controls; instead, we used Cavaco et al. (2008b, 2013b) as a reference. For VFT, the depressed groups did not differ in semantic fluency (animals) (*t*(36) = -.816, *p* = .420, *d* = -.263) or phonemic fluency (M–R–P) (*t*(35) = -.109, *p* = .914, *d* = -.035). For AVLT, no differences were found between the patient groups in terms of immediate recall (*t*(37) = -1.007, *p* = .321, *d* = -.327) delayed recall (*t*(37) = -1.603, *p* = .117, *d* = -.514), or memory retention (*t*(37) = -.504, *p* = .617, *d* = -.160) (Figure 1).

## Discussion

Some of the results of our study are atypical. Our results are in line with those of King et al. (2000), who challenged the widely discussed theory that depressed suicidal individuals are usually characterized by EF deficits, but they conflict with most reports (Jollant et al., 2011; Keilp et al., 2001, 2008, 2013; Marzuk et al., 2005; Richard-Devantoy, Gorwood, et al., 2012; Richard-Devantoy, Jollant, et al., 2012; Richard-Devantoy et al., 2014; Westheide et al., 2008). In our study, both groups of depressed patients were outperformed by healthy controls on most tests assessing EFs, as reported in previous studies (Beats et al., 1996; Biringer et al., 2005; Jollant et al., 2011; Keilp et al., 2001, 2008, 2013; King et al., 2000; Lee et al., 2012; Marzuk et al., 2005; McDermott & Ebmeier, 2009; Richard-Devantoy, Gorwood, et al., 2012; Richard-Devantoy, Jollant, et al., 2012; Richard-Devantoy et al., 2014; Rogers et al., 2004; Roiser et al., 2009; Stordal et al., 2004; Wagner et al., 2012; Westheide et al., 2008), and the results were statistically significant for TMT part B, IGT, and WCST (perseverative errors and conceptual-level responses). This finding confirms our first hypothesis (i.e., that all depressed subjects will perform worse than non-depressed subjects).

Similarly, our results do not provide sufficient evidence to clearly differentiate between depressed attempters and depressed non-attempters. Our suicidal group did not perform worse than depressed patients who had never attempted suicide in, measures such as WCST or IGT, which are widely recognized to provide important evaluations regarding set shifting and decision making. Therefore, our second hypothesis (i.e., that depressed suicide attempters will generally exhibit more executive deficits than depressed non-attempters and healthy controls) is not confirmed. When we isolated only the suicidal subjects and compared performance based on the suicidal act (violent and non-violent), we did not find differences, particularly in IGT, as reported in the recent study of Wyart et al. (2016). This finding may relate to the size of the suicidal group in our study; in the future, the sample size should be increased to improve statistical power and to control the type of suicide attempt.

A surprising result in our study is the difference in the results for TOL (extra moves). In contrast to depressed non-attempters, depressed attempters’ performances on TOL did not differ from those of healthy controls, which may indicate that their planning ability was relatively intact at the time of assessment. As in previous studies (Beats et al., 1996; Elliott et al., 1996), we expected poor performances in both depressive groups, and this normative performance in planning might contribute to the risk of suicide because it enables organization of each step in pursuit of a certain objective or intention (Lezak, Howieson, & Loring, 2004), such as a suicidal act. We could not, however, exclude a false positive in this last result. If we made a Bonferroni adjustment, dividing the alpha value (.05) by the number of tests (9), we would obtain a new p-value of .00055. Therefore, the TOL result is not significant.

As with the subjects in Westheide et al. (2008) and Richard-Devantoy, Ding, Lepage, Turecki, and Jollant (2016), the depressed attempters were clearly distinguishable from the non-attempters and healthy controls through the former’s deficit in response inhibition and relatively poor performance in relation to GNG-commission errors. The influence of cognitive inhibition on the cognitive functioning of depressed suicide attempters must be further investigated to improve our understanding of the potential impairments related to suicidal behavior and the benefits of cognitive rehabilitation, for example training-induced behavioral and brain plasticity in inhibitory control (Spierer, Chavan, & Manuel, 2013).

### Conclusions

The results of the current study distinguish between depressed attempters and depressed non-attempters based on their performance in response inhibition, which is essential for selecting the behavior needed to respond appropriately or to inhibit an inappropriate response (Simmonds, Pekar, & Mostofsky, 2008). Furthermore, we must acknowledge the presence of normative planning, which may allow subjects to order and prioritize ideas essential to the development of a conceptual scheme to carry out a determined plan (Lezak et al., 2004), such as a suicidal plan.

Finally, executive functioning deficits appear to be related to the depressive state. In our sample, no difference was found in the performance of the two groups of depressed subjects (suicidal and non-suicidal), particular in the classic neuropsychological assessment (e.g., IGT, WCST, VST, TMT).

To better understand these findings, our study should be replicated using larger samples.
